# Comparison of students' readiness from six health education programs for interprofessional learning in Vietnam: a cross-sectional study

**DOI:** 10.1186/s12909-023-04776-2

**Published:** 2023-10-25

**Authors:** Nguyen Thi Thanh Huyen, Nguyen Minh Tam, Johan Wens, Giannoula Tsakitzidis, Le Van Chi, Le Ho Thi Quynh Anh, Che Thi Len Len, Huynh Van Chuong, Nguyen Vu Quoc Huy, Martin Valcke

**Affiliations:** 1 Family Medicine Centre, Hue University of Medicine and Pharmacy, Hue University, Hue, Vietnam; 2 Department of Primary and Interdisciplinary Care, Faculty of Medicine and Health Sciences, University of Antwerp, Antwerp, Belgium; 3 Department of Internal Medicine, Hue University of Medicine and Pharmacy, Hue University, Hue, Vietnam; 4 The Education Quality Management Agency, Ministry of Education and Training, Hanoi, Vietnam; 5 Department of Obstetrics & Gynaecology, Hue University of Medicine and Pharmacy, Hue University, Hue, Vietnam; 6 Department of Educational Studies, Faculty of Psychology and Educational Sciences, Ghent University, Ghent, Belgium

**Keywords:** Interprofessional education, Interprofessional learning, Student’s readiness, Undergraduate healthcare students

## Abstract

**Background:**

Interprofessional education (IPE) is expected to help prepare undergraduate health profession students to collaborate with other healthcare professionals in realising quality of care. Studies stress the necessity of students’ readiness for interprofessional learning (IPL) in view of designing IPE programs. The present study aims to determine students' IPL-readiness and looks at related differences in students enrolled in different programs and at different phases in their educational program.

**Methods:**

A cross-sectional survey study was set up among 1139 students from six health programs at HueUMP, using the Readiness for Interprofessional Learning Scale (RIPLS). Statistical analysis was performed using Kruskal–Wallis H and Mann–Whitney U tests.

**Results:**

The overall mean RIPLS score was 68.89. RIPLS scores significantly differed between programs and between phases in the educational programs. Medical students presented a lower readiness level for IPL than students from other programs. In contrast to a significant increase in RIPLS scores of students in the clinical phase in Vietnamese traditional medicine, medicine, and pharmacy, a decrease in RIPLS scores was observed in students in the clinical phase in odonto-stomatology.

**Conclusions:**

The differences could be related to differences in educational programs and the study phases in a particular program. These results offer insights to direct the design and implementation of IPE in health education curricula and especially underscore the need to provide IPE throughout the curriculum.

## Background

Working effectively in a healthcare team, communicating productively and understanding each other’s roles helps improve the quality of care of patients [1, 2]. Interprofessional education (IPE) is reported to prepare undergraduate health profession students to collaborate with other healthcare professionals in realising quality of care [3, 4]. Interprofessional learning (IPL) offers students opportunities to work together with and learn from and about other professions [5].

IPE programs have been found to be implemented in many universities. At the same time, related research results show the need to strengthen IPE [6–8]. However, in Asian countries, IPE implementation has received little attention [9]. In Vietnam – the country focused upon in this article—Huyen and colleagues reported a lack of interprofessional collaboration between primary healthcare providers in daily work and how this could be related to the lack of IPE training [10]. Out of 29 medical universities in Vietnam, only one implemented IPE in the undergraduate curriculum [11, 12]. As one of the larger medical universities in Vietnam, the University of Medicine and Pharmacy, Hue University (HueUMP) took the initiative to design, develop and implement an IPE module in its health and medical science-related curricula.

Student involvement is a key factor in establishing a successful interprofessional education [13]. This can be operationalised by studying students’ 'Readiness for interprofessional learning'. Readiness is considered a precursor of a student's intention and willingness to participate in the IPL [14]. Available research points at the relevance of looking at students’ IPL-readiness in view of designing IPE programs and related learning activities [15, 16]. Nevertheless, in the literature, little is found about students' readiness for IPL when comparing professions and how this readiness evolves during an educational program. This brings us to the general research problem of the present study, set up in the Vietnamese context: what is the status of students’ IPL readiness from different educational programs? Related research question focuses on between-program differences and within-program differences when comparing students at the start and the end of their study program. Therefore, we conducted this study to determine students' IPL readiness and the potential differences in IPL-readiness when comparing students between health programs and within programs when focusing on changes between early and later phases in student careers at HueUMP. The findings are expected to guide IPE design and implementation in further research.

## Methods

### Design

A cross-sectional quantitative survey design was adopted [17]. This allows for studying a large population with limited recourses [18].

### Study setting

The study was set up at the University of Medicine and Pharmacy, Hue University. Students enrolled in six of the ten undergraduate training programs of HueUMP were invited to participate: medicine, odonto-stomatology (OS), preventive medicine (PM), Vietnamese traditional medicine (VTM) with a program of six years, pharmacy with a program of five years, and nursing with a program of four years. IPE is not yet an integral part of education at HueUMP. Clinical rotations start in the third year in each of the six programs, during which collaboration with other professionals is not yet organised systematically. Building on a policy decision to switch from a uni-professional to an IPL-infused curriculum, curriculum redesign will start after developing an in-depth understanding of students’ IPL-readiness in each of the six programs mentioned above.

### Study population and sample

Considering the differences in the duration of the different programs, sampling was based on the following stratification variable: either enrolled in the pre-clinical phase or enrolled during the clinical phase of the program. This resulted in inviting students from the second and fifth years enrolled in Medicine, OS, PM and VTM and pharmacy students enrolled in the second and fourth years; nursing students from the second and third years were asked to participate. The Undergraduate Office of HueUMP provided a list of classes and student numbers for each class in six programs. Half of all classes in each program in the second and the penultimate year were selected randomly (16 out of 32 classes and 1160 out of 2282 students (51.0% of the population). This can be considered as a representative sample of the population being studied [19].

### Instrument

The research instrument consisted of two sections. The first section aimed at collecting demographic information (sex, age, health education program, current year of study, and previous participation in any IPE courses). The second section used the Readiness for Interprofessional Learning Scale (RIPLS) to assess students' IPL readiness. The RIPLS is a 19 items tool consisting of four subscales: 'teamwork and collaboration' (TC) (items 1–9), 'negative professional identity' (NPI) (items 10–12), 'positive professional identity' (PPI) (items 13–16), and 'roles and responsibilities' (RR) (items 17–19) [20]. Respondent uses a 5-point Likert scale to reply to statements (1 = strongly disagree, 5 = strongly agree). The total RIPLS score ranges from 19 to 95. A psychometric study of the most recent version of the instrument underpins its reliability and validity [21]. The reliability and validity of the RIPLS scale have further been assessed in a range of cultural contexts, such as in China [22], in Japan [23], in Spain [24], in Turkey [25], and in United Kingdom [26]. However, psychometric information about the RIPLS in the Vietnamese context is unavailable. Preliminary analyses will therefore be carried out to document instrument quality. The RIPLS was translated using the back translation method to ensure consistency between the original and translated versions of the RIPLS [27].

### Data collection

The data were collected between May 12 and May 22, 2022. Participants were invited to a classroom to fill out the survey. All participants were informed about the study's objective and signed an informed consent form. It took approximately 15 min to complete the research instrument.

### Quality of the Vietnamese version of the RIPLS

The internal consistency of the total RIPLS was 0.78. Cronbach alpha values for the subscales were good for TC (α = 0.81), NPI (α = 0.84), and PPI (α = 0.77) but low for RR (α = 0.52). The low reliability of the 'roles and responsibilities' subscale is not unexpected since the three roles, as reflected in the three subscale items, represent different valid sets of responsibilities in a professional. Adopting one specific responsibility/role does not automatically imply that the other roles/responsibilities are also being adopted. The focus on the different roles/responsibilities underpins the content validity of this subscale, but this is at the expense of its reliability. The weak internal consistency was already identified by the original scale authors [20]. This observation also explains why in comparable RIPLS research, not all subscales were included in the studies [28] or why low-reliability scores for this subscale were reported [29]. Pearson's correlations show significant correlations between the four subscales (between *r* = 0.594 and *r* = 0.001), pointing at a relative independence of the four subscales.

### Data analysis

Data analysis was carried out with Statistical Package for the Social Sciences (SPSS, version 28.0). The results were considered statistically significant if *p* < 0.01. The coding was reversed for the NPI and RR subscales to guarantee that higher scores reflect that students are more ready for IPL in the four subscales [29, 30]. The preliminary analysis focused on the quality of the instrument (scale statistics and factor analysis). Next, descriptive statistics were calculated, and basic comparisons were carried out on the base of the demographic variables.

Next, the mean RIPLS scores were compared between and within the different health education programs. Since the (sub)scale scores were not normally distributed (Kolmogorov–Smirnov test *p* < 0.05). Based on the valid RIPLS, the Kruskal–Wallis H test was used to compare the RIPLS scores for different health education programs. Mann–Whitney U test was performed to analyse within-program differences. When carrying out multiple comparison tests to see which groups are different from the others, a Bonferroni correction for significance was applied.

## Results

Out of 1160 invited students, 1139 students (454 medicine, 198 nursing, 192 pharmacy, 85 PM, 92 VTM, and 118 OS students) participated and completed the questionnaires, representing an overall response rate of 98.2%. Table 1 summarises the demographic characteristics of all students in the different health science programs. The final sample consisted of 66.5% female students (*n* = 758) and 33.5% male students (*n* = 381). The average age of the participants was 21.24 (± 1.59). Five hundred sixty-six second-year students in the pre-clinical phase of the educational program participated in the study (49.7%). In total, 573 penultimate-year students in the clinical phase were involved (50.3%).

**Table 1:** Demographic characteristics of participants (*N* 1139)

	Total	Medicine	Nursing	Pharmacy	Preventive Medicine	Vietnamese traditional medicine	Odonto-Stomatology
**Sex [n (%)]**							
Male	381 (33.5)	229 (50.4)	15 (7.6)	41 (21.4)	24 (28.2)	26 (28.3)	46 (39.0)
Female	758 (66.5)	225 (49.6)	183 (92.4)	151 (78.6)	61 (71.8)	66 (71.7)	72 (61.0)
Total	1139 (100.0)	454 (100.0)	198 (100.0)	192 (100.0)	85 (100.0)	92 (100.0)	118 (100.0)
**Age [mean (SD)]**							
	21.24 (1.59)	21.69 (1.72)	20.36 (0.71)	20.88 (1.19)	21.84 (1.79)	21.4 (1.8)	21.43 (1.64)
**Phage of study [n (%)]**							
Pre-clinical phase	566 (49.7)	210 (46.3)	101 (51.0)	102 (53.1)	40 (47.1)	52 (56.5)	61 (51.7)
Clinical phase	573 (50.3)	244 (53.7)	97 (49.0)	90 (46.9)	45 (52.9)	40 (43.5)	57 (48.3)

Table 2 summarises mean and standard deviation values for the four subscales and the total RIPLS scores. Additionally, this table displays the mean RIPLS scores resulting from the multiple comparisons of the six programs and the comparison between the early and final phases in each program. The overall mean RIPLS score was 68,89 (SD ± 6,08). No significant differences are observed in the total RIPLS score of students between programs and between the phase in their study programme.

**Table 2:** Multiple comparisons of students' mean RIPLS scores between six programs and between years of study

		Medicine [X_mean_ ± SD]	Nursing [X_mean_ ± SD]	Pharmacy [X_mean_ ± SD]	Preventive Medicine [X_mean_ ± SD]	Vietnamese traditional medicine [X_mean_ ± SD]	Odonto-Stomatology [X_mean_ ± SD]	***p*-value**
		*n* = 454	*n* = 198	*n* = 192	*n* = 85	*n* = 92	*n* = 35	
**Total RIPLS score**								
Phage of study	Pre-clinical phase	68.05 ± 5.55	67.76 ± 5.73	68.16 ± 5.87	68.48 ± 7.65	66.35 ± 7.32	69.64 ± 6.49	0.196
	Clinical phase	68.43 ± 5.90	68.90 ± 6.57	69.28 ± 6.45	66.29 ± 7.37	69.13 ± 6.97	66.47 ± 7.87	0.356
***p*-value**		0.542	0.706	0.150	0.236	0.109	0.029	
Total^*^		68.25 ± 5.74	68.32 ± 6.17	68.68 ± 6.16	67.32 ± 7.54	67.55 ± 7.21	68.11 ± 7.33	0.289
**Teamwork and collaboration (TC)**								
Phage of study	Pre-clinical phase	37.21 ± 3.63	36.13 ± 3.09	37.39 ± 3.99	36.55 ± 4.13	37.04 ± 4.35	36.64 ± 3.47	0.075
	Clinical phase	36.82 ± 3.37	36.36 ± 4.04	36.68 ± 3.45	35.04 ± 4.21	36.85 ± 3.79	36.26 ± 4.79	0.063
***p*-value**		0.245	0.706	0.311	0.078	0.475	0.901	
Total^*^		37.00 ± 3.50	36.24 ± 3.58	37.06 ± 3.75	35.75 ± 4.21	36.96 ± 4.09	36.46 ± 4.15	**0.004**
**Negative professional identity (NPI)**								
Phage of study	Pre-clinical phase	10.86 ± 2.56	10.67 ± 2.32	10.71 ± 2.98	11.28 ± 2.37	9.35 ± 2.90	11.31 ± 2.55	** < 0.001**
	Clinical phase	11.15 ± 2.14	11.44 ± 1.90	11.48 ± 2.15	11.09 ± 2.20	11.05 ± 2.63	10.30 ± 2.99	0.228
***p*-value**		0.549	0.029	0.188	0.720	**0.005**	0.052	
Total^*^		11.01 ± 2.35	11.05 ± 2.15	11.07 ± 2.64	11.18 ± 2.27	10.09 ± 2.90	10.82 ± 2.81	0.033
**Positive professional identity (PPI)**								
Phage of study	Pre-clinical phase	15.97 ± 1.94	15.97 ± 1.68	15.92 ± 2.20	15.98 ± 2.35	16.04 ± 2.36	15.87 ± 1.93	0.968
	Clinical phase	15.60 ± 2.09	15.64 ± 2.45	15.82 ± 2.06	15.33 ± 2.44	16.50 ± 1.80	15.44 ± 1.88	0.201
***p*-value**		0.022	0.206	0.764	0.344	0.880	0.173	
Total^*^		15.77 ± 2.02	15.81 ± 2.09	15.88 ± 2.13	15.64 ± 2.40	16.24 ± 2.13	15.66 ± 1.91	0.263
**Roles and responsibilities (RR)**								
Phage of study	Pre-clinical phase	7.94 ± 1.72	9.02 ± 1.71	8.07 ± 2.16	8.70 ± 1.77	8.10 ± 1.81	9.70 ± 1.71	** < 0.001**
	Clinical phase	8.76 ± 1.87	9.31 ± 1.72	9.24 ± 1.52	8.69 ± 1.31	8.88 ± 2.11	8.33 ± 1.85	**0.006**
***p*-value**		** < 0.001**	0.293	** < 0.001**	1.000	0.069	** < 0.001**	
Total^*^		8.38 ± 1.84	9.16 ± 1.71	8.62 ± 1.97	8.69 ± 1.54	8.43 ± 1.97	9.04 ± 1.90	** < 0.001**

^*^Total population in each program

The Kruskal–Wallis H test results revealed a statistically significant difference in the TC subscale (*p* = 0.004) and the RR subscale score (*p* < 0.001). Multiple pairwise comparison analysis was carried out, applying Bonferroni correction for significance. This clarified that no significant difference was to be found in the TC subscale between any of the two programs.

However, in the RR subscale, pairwise comparison analysis showed that the mean RIPLS score of nursing students was significantly higher than that of medical students (*p* < 0.001). Also, the mean RIPLS score of OS students was significantly higher than that of medical students (*p* = 0.007). The highest mean RR RIPLS score was observed in nursing (9.16 ± 1.71) and OS students (9.04 ± 1.90). Medicine students reported the lowest mean RR RIPLS scores (8.38 ± 1.84).

Focusing on students in the pre-clinical phase, the Kruskal–Wallis H test results point at significant differences in relation to the NPI (*p* < 0.001) and the RR subscale (*p* < 0.001). Pairwise comparison analysis, after the Bonferroni correction, the NPI scores were significantly higher in the pre-clinical phase OS students as compared to VTM students (*p* = 0.001). Also, medical students' mean RIPLS scores were significantly higher than those in VTM (*p* = 0.007).

In the RR subscale, the mean RIPLS scores of OS students in the clinical phase were significantly higher than that of students in VTM (*p* < 0.001), in pharmacy (*p* < 0.001), and in medicine (*p* < 0.001). Nursing students' mean RIPLS scores were significantly higher than medical students (*p* = 0.001). For students in the clinical phase, a statistically significant difference was only found in the mean RR scores when comparing students of the six programs (*p* = 0.006). However, pairwise comparison analysis, after Bonferroni correction, indicated no statistically significant difference in the mean RIPLS scores of students between any two professions.

Looking at differences between students in the pre-clinical and clinical phases, the Mann–Whitney U statistical analysis results pointed at differences in students enrolled in Vietnamese traditional medicine (VTM) in their NPI RIPLS scores. VTM students in the pre-clinical phase scored significantly lower than students in the clinical phase (*p* = 0.005). The RR subscale scores mirror a more complex picture. Both in medicine and pharmacy, the NPI scores of students in the clinical phase were significantly higher than the scores of students in the pre-clinical phase (*p* < 0.001). Nevertheless, in Odonto-Stomatology students, students in the pre-clinical phase outperform students in the clinical phase (*p* < 0.001).

## Discussions

This study aimed to map the IPL-readiness of students enrolled in six health programs and to study differences between educational programs and within these educational programs.

In this study, the relatively large sample size and the high response rate provided a high confidence level to arrive at robust results [31]. Students in the pre-clinical and clinical phases had a similar participation rate in the total population and each educational program. This supports the representation of the population in the pre-clinical and clinical phases of the educational program [17].

The results reveal that – in this Vietnamese university context—the total RIPLS scores of students hardly changed over the years in their educational program. This is to be expected considering the lack of a focus on IPE in the current curriculum of all health education programs. It is also noted that involvement in internships (students in the clinical phase) did not automatically lead to developing interprofessional competence, which does not seem to develop implicitly. An explicit focus is deemed necessary.

Looking at the RIPLS subscales, some differences could be detected. The NPI score is the lowest in Vietnamese Traditional Medicine students in the pre-clinical phase, suggesting that these students are less ready for IPL than students in other programs. This could be related to the nature of the educational program that initially builds mainly on specific therapies by drugs composed of herbs, acupuncture, manual therapies, and spiritual practices [32]. However, traditional medicine usually interacts with Western Medicine in the Vietnamese healthcare context [33]. This is also the policy of the Vietnamese Ministry of Health [34]. From the third year in the VTM program, students acquire fundamental conceptions of Western medicine and do clinical rotations in some Western medicine departments, including internal, external, paediatric, and Obstetrics & Gynaecology medicine. This is mirrored in VTM students in the clinical phase, who report significantly higher RIPLS scores.

In the discussion about the Roles and Responsibilities subscale, the reliability analysis pointed at the particular nature of this scale. This should be considered when interpreting the following findings. Medical students reported the lowest RR scores compared to students from other programs. This suggests that medical students adopt a wider range of roles and responsibilities to a lesser extent than students in other programs. Also, other studies found lower RIPLS scores in medical students as compared to other health education programs [26, 35]. Additionally, Oliviera et al. showed that nursing, dentistry and pharmacy degree course students mirrored higher RIPLS scores than medicine students [36]. This could be explained by the stronger confrontation with other professions in the non-medicine programs [37]. This may also be influenced by the strong social hierarchy culture in the medical profession in Asian regions [38]. This confirms earlier research findings that uncovered a considerable power distance between medical doctors and other professions, including nurses, midwives, and physician assistants, in Vietnamese clinical practice settings [10]. These culture-related differences cannot be neglected, even when sharing roles and responsibilities seems a good idea from an IPE perspective.

In view of developing educational programs, the analysis focusing on students in the pre-clinical and clinical phases is key. In contrast to our expectations, students hardly evolve in their IPL readiness. Only in relation to the Roles and Responsibilities subscale we find significant differences when students evolve over time. The findings in relation to the medical and pharmacy students who mirror an increased awareness of their roles and responsibilities were also noted in other studies and were explained by the exposure to a larger number of clinical subjects and settings in their curriculum [36, 39]. Additionally, during clinical rotations, these students got more opportunities to observe the daily work in a range of clinical departments to understand the roles and responsibilities of other professions. Also, students in the clinical phase get opportunities to build up a social network with students from other programs. This points at conditions to optimise the potential to develop IPE in students during all phases of an educational program. For OS students in the clinical phase, RR-related readiness seems to decrease. This could be related to OS students who hardly interact with other professions or either interact with other professions in a particular way; e.g., the interaction with nurses remains limited to receiving their support (see also [36, 40]. In the Vietnamese clinical context, OS experts usually work in a one-to-one setting with a patient, and when a nurse is present, this is solely to support the dentist’s actions. This could also be linked to the tendency to have a hierarchical attitude in students in the clinical phase who consider doctors being more important than nurses. This attitude negatively affects interprofessional collaboration within healthcare teams.

These particular results, especially the observation that IPL readiness hardly changes during health study careers, confirm that the implementation of IPE in higher education, especially at HueUMP, is urgently needed [4]. Besides, these findings inspired HueUMP to develop and implement a novel IPE module for students during their clinical phase [41, 42]. The IPE module gives students from different health educational programs opportunities to learn from, about and with each other; not only in the classroom but in the clinical workplace context. Within clinical practice learning activities, students will collaborate with students of other programs in an authentic IPC setting to cater for morbidity patients in a primary care context [43]. As a result, students are expected to improve their readiness for IPL and their IPC competencies, resulting in better health care provision for patients.

## Limitations

A limitation of the present study is related to its cross-sectional design, which is less effective in mapping the longitudinal development of latent variables in student cohorts within the same educational program. Also, the study builds on quantitative data that could be enriched with qualitative data to develop a more in-depth understanding of the mechanisms that are responsible for the differences being uncovered. An additional limitation is the double amount of female students compared to male students in our sample. This can be explained by the higher enrolment number of female students in medical universities, especially in the nursing and pharmacy programs.

## Conclusions

The present study observed differences in students' Readiness for interprofessional learning. The differences could be related to differences in educational programs and the study phases in a particular program. Medical students presented a lower readiness level for interprofessional learning compared to students from other programs. Also, no overall significant increase in readiness for IPL was observed when looking at later phases in a program. These results offer insights to direct the design and implementation of IPE in health education curricula and especially underscore the need to provide IPE throughout the curriculum. Furthermore, considering the lack of attention paid to IPE in medical universities in Southeast Asia countries and beyond, the present study provides health education administrators with an overview of the actual IPL-readiness of students enrolled in different programs. This is expected to result in more attention being paid to the development and implementation of IPE in their curricula.

## Data Availability

The datasets used and analysed during the current study are available from the corresponding author upon reasonable request.

## Abbreviations

IPE: Interprofessional education
IPL: Interprofessional learning
HueUMP: Hue University of Medicine and Pharmacy, Hue University
OS: Odonto-Stomatology
PM: Preventive medicine
VTM: Vietnamese traditional medicine
RIPLS: Readiness for Interprofessional Learning Scale
TC: Teamwork and collaboration
NPI: Negative professional identity
PPI: Positive professional identity
RR: Roles and responsibilities
WHO: World Health Organization
